# Chromatin dynamics and cellular energy homeostasis

**DOI:** 10.1093/jb/mvag051

**Published:** 2026-07-01

**Authors:** Tamaki Suganuma, Jerry L Workman

**Affiliations:** Stowers Institute for Medical Research, Workman Lab, 1000 E. 50th Street, Kansas City, MO 64110, USA; Stowers Institute for Medical Research, Workman Lab, 1000 E. 50th Street, Kansas City, MO 64110, USA

**Keywords:** chromatin, histone modification, metabolism, nucleosome, transcription

## Abstract

Metabolism is a complex consortium of chemical reactions, consuming and releasing energy. In addition, cellular respiration is a pivotal catabolic process of nutrients using oxygen to generate ATP. Dynamics of chromatin structure are fundamental to genomic events in all cellular processes. Therefore, chromatin consumes robust cellular energy formed in metabolism. To regulate cellular processes, chromatin functions and metabolism need to be tightly coordinated in response to environmental changes including nutrition availability. Indeed, molecular oxygen directly impacts DNA and chromatin modification and influences cell fate. Herein, we review fundamentals of chromatin dynamics, nucleosome modifications and roles of chromatin dynamics in spatiotemporal regulation of cellular energy homeostasis.

## Graphical Abstract

**Graphical Abstract:**
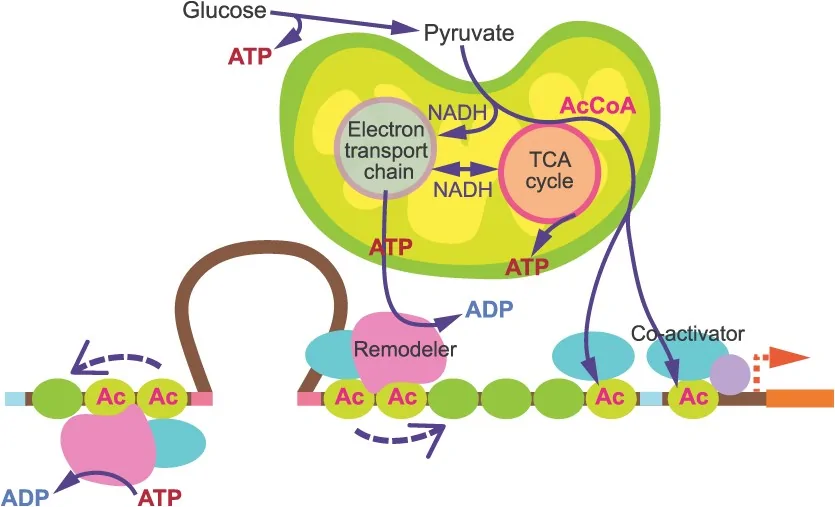

Oxygen is an important molecule for eukaryotic life. In aerobic respiration, 90 to 95% of oxygen consumed in the body is used in mitochondria to generate cellular energy in the form of ATP (*1**,*  *2*). ATP is in high demand for macromolecule biosynthesis including DNA and RNA synthesis (*1**,*  *3**,*  *4*). Oxygen has a high standard reduction potential; therefore, it readily oxidizes other substances. Overproduction of intermediate species of oxygen, reactive oxygen species (ROS), can be harmful for the structures of cells and tissues including lipids, membranes, proteins and DNA through disruption of redox events (*1**,*  *5**,*  *6*). At low or moderate concentrations, however, ROS turn out to be beneficial for obtaining immunity in individuals through their antimicrobial activity and as signalling molecules for oxidative stress responses (*7**,*  *8*). Anaerobic respiration produces fewer ROS compared to aerobic respiration (*9*). Anaerobic species or tissues have alternative respiratory chain redox systems; however, they still produce ROS that damage proteins and DNA (*9*).

Metabolism is the sum of all chemical reactions in energy anabolic and catabolic processes and maintains energy homeostasis. Cellular respiration is a metabolic process generating ATP from glucose (*10*). Hence, metabolism influences cell fate by consuming molecular oxygen. Chromatin greatly consumes cellular energy by its dynamics including nucleosome modification. Therefore, coordination between chromatin functions and metabolism in response to environmental changes and cellular metabolic status is pivotal to establishing coherent energy homeostasis. Imbalanced energy distribution by disruption of this coordination leads to developmental malfunctions, disorders including neurodegenerative disease and tumourigenesis (*3**,*  *11–13*).

Here, we review roles of chromatin dynamics in redox and energy homeostasis by recapturing features of (i) chromatin structure dynamics and nucleosome modification, (ii) fundamental functions of chromatin modifiers, (iii) the interplay between metabolite, molecular oxygen and chromatin dynamics, and (iv) chromatin dynamics in spatiotemporal events that result in transcription. We will also discuss metabolic changes impacting variations in DNA sequence, in which inappropriate changes may interrupt the process of essential chromatin dynamics by altering the nucleosome substrate itself.

**Fig. 1:**
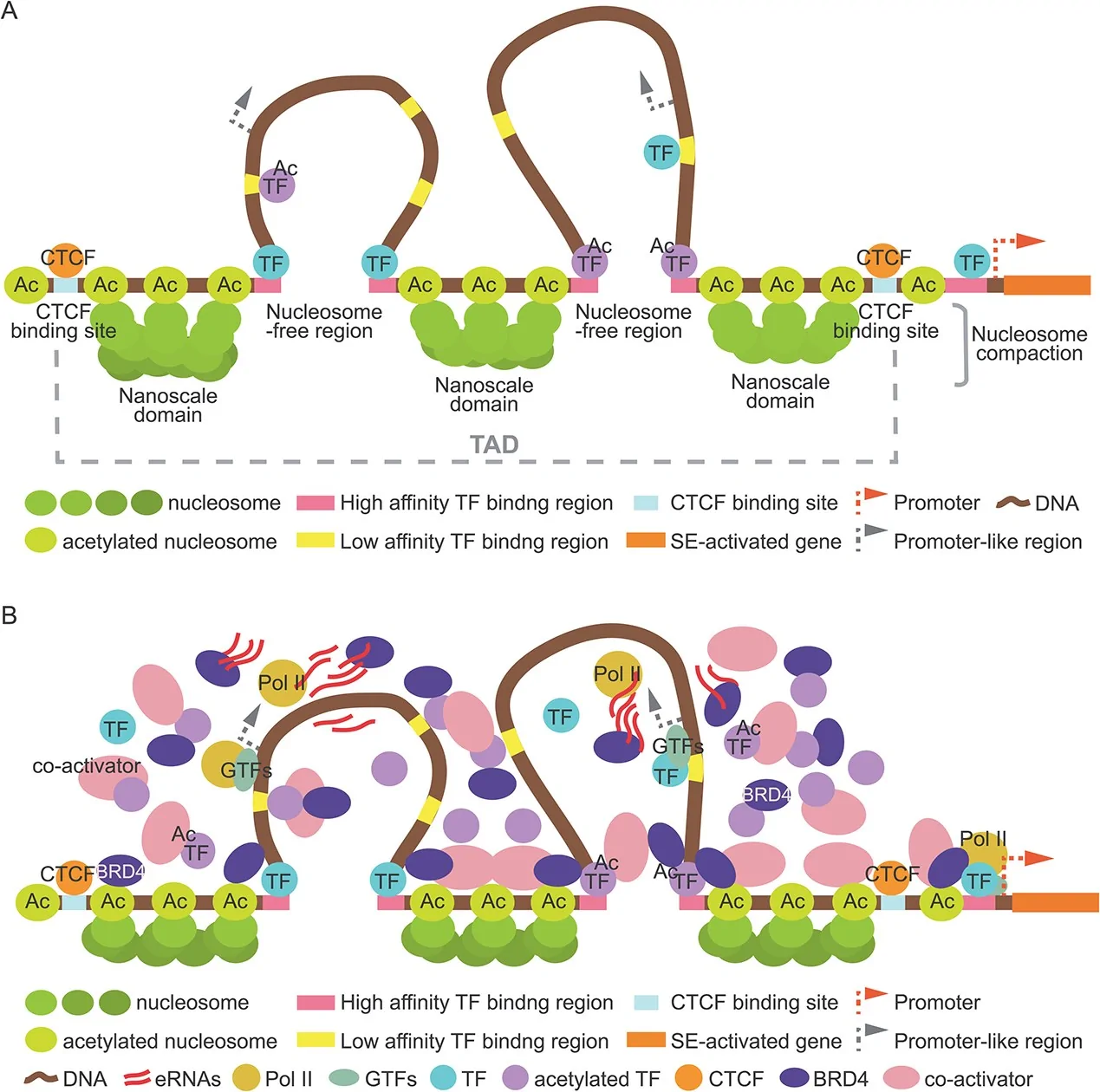
**Summary model of TAD formation and chromatin dynamics in SE creation. (**A) There are high-compacted nucleosomes and nucleosome-free regions within CTCF organized TADs. The nucleosome-free regions extend above the chromatin surface. Regions flanking the nucleosome-free regions are compartmentalized with compacted nucleosomes of which surfaces are enriched in H3K27-acetylated nucleosomes that form relaxed chromatin. To create nucleosome-free regions, which are critical for transcription activation, TFs bound to regions flanking nucleosomes may need to act as barriers to nucleosome sliding activity (Fig. 2). Sequence variations may lead to preferences of enhancers in enhancer-enhancer connections, which create loop formation (Fig. 2). (B) Our proposed model for creations of SE is illustrated. TFs’ low affinity regions do not contribute to maintaining nucleosome-free regions; however, these regions may contribute to eRNA transcription to function as promoter/enhancer-like regions in DNA loops that are partitioned between nanoscale nucleosome domains (A). Acetylated nucleosomes, acetylated TFs and eRNA are BRD4 bromodomain binding substrates. The high density of these macromolecules generates CTCF-mediated SE using phase separation. GTFs, general TFs.

## Nucleosomes are key substrates in chromatin dynamics and cellular energy homeostasis

For proper cellular processes, chromatin regulators and cellular fitness need to be coordinated. Details of these complex mechanisms are built with chromatin as a substrate and contain many components, which regulate nucleosomes. Chromatin is composed of repeating units of nucleosomes. The nucleosome comprises a histone octamer consisting of two copies of each core histone H2A, H2B, H3 and H4, wrapped by 147 base pair (bp) of double-stranded DNA. In humans, there are eight variants of H2A, six variants of H3 and two testis-specific variants of H2B. Many histone variant genes lack introns, and their mRNAs are not polyadenylated as with canonical core histones (*5**,*  *14*). These histone genes encode histone protein expressed during S-phase for packaging newly synthesized DNA into chromatin (replication-dependent histones) (*15*). By contrast, the genes of some other histone variants including CENPA and macro-H2A contain introns and their mRNAs are polyadenylated (*15*). Synthesis of these histones is replication independent (replication-independent histones) (*5**,*  *15*). Preferences of histone chaperones for binding to distinct histones contribute to proper assembly or disassembly of nucleosomes (*5*). After DNA replication, ATP-dependent chromatin remodelling complexes regularly space nucleosomes as a part of the chromatin assembly process (*16**,*  *17*). During aging, however, increased replication-independent nucleosome assembly leads to more ectopic and fuzzy nucleosome positioning (*5*).

ATP-dependent chromatin remodelling complexes are classified into four families: imitation switch (ISWI), chromodomain helicase DNA-binding (CHD), switch/sucrose non-fermentable (SWI/SNF) and INO80 (*18–23*). The common mechanisms among ISWI, CHD and SWI/SNF are that they use ATP hydrolysis for executing DNA translocation from a fixed location, two DNA helical turns away from the nucleosome dyad (*18*). Nucleosome spacing is primarily determined by the ISWI and CHD subfamily remodellers (*18**,*  *24*). ISWI binds linker DNA and then slides ~1 bp of a nucleosome per ATP hydrolysis (*18**,*  *25*). Repeating the action draws a nucleosome to abut the adjacent nucleosome, establishing the next nucleosome at a fixed distance from the substrate nucleosome (*18*). There are two homologs of ISWI in yeast, ISW1 and ISW2, forming distinct complexes (*26*). Interestingly, ISW2 binding distorts the nucleosome before the ATPase motor moves on nucleosomal DNA (*27*). In contrast, the yeast SWI/SNF subfamily member, RSC complex, moves along one of the two strands of the DNA, moving in the 3′–5′ direction, leading to one helical rotation of DNA for every ∼10 bp that is translocated (*28*). RSC remodelling utilizes higher ATPase activity and coupling efficiency (translocated DNA base-pairs per ATP hydrolysis cycle), changing the nucleosome remodelling reactions from nucleosome sliding to histone ejection (*29**,*  *30*). The common function of remodelling complexes is that the DNA propelling action of remodellers moves 1–2 bp of DNA per cycle of ATP binding–hydrolysis (*18*). Hence, ATP-dependent chromatin remodelling complexes can alter the chromatin structure to both transcriptional active and silent status by utilizing ATP.

**Fig. 2:**
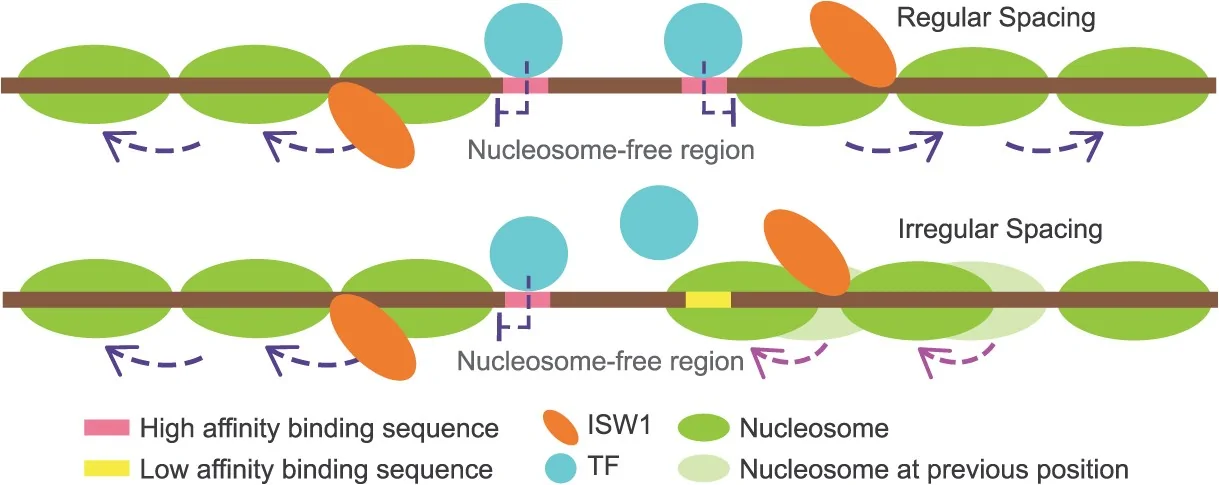
**Model illustrating the establishment of nucleosome-free regions based on DNA sequence variations in TFs- and CTCF-binding regions flanking regions of nucleosomes.** ISW1 nucleosome sliding is important to maintain nucleosome-free regions (*24*). DNA binding of TFs to their binding regions are affected by variations in their binding sequences and compete with ISW1 sliding activity. The amount of TFs does not affect ISW1 activity *in vitro* (*40*), suggesting creation of nucleosome-free regions is strongly influenced by sequence of DNA motifs.

ATP-dependent chromatin remodelling complexes play crucial roles in every level of chromatin organization. Even the organization of three-dimensional (3D) structures within large chromatin domains, topologically associated domains (TADs), is dependent on ATP-dependent chromatin remodellers (*31–33*) (Fig. 1A). TADs are characterized by a high frequency of intra-domain chromatin contact and play decisive roles in gene expression (*34*). A recent study of chromatin conformation capture (3C) mapping at base-pair resolution used a novel approach termed ‘Micro Capture-C ultra’ (MCCu). This study revealed that nucleosome-associated chromatin (densely condensed nucleosomes) forms nanoscale domains of interactions, within conventional TADs, which are interrupted by nucleosome-free regions (*35*) (Fig. 1A). The nucleosome-free regions extend above the chromatin surface, potentially forming phase-separated condensates that act as hubs for abundant transcription factors (TFs) and co-activators (*35*) (Fig. 1B). Using MCCu, it was found that the contacts between enhancers and promoters are facilitated by the nucleosome-free regions in each enhancer and promoter as well as by enhancer and promoter regulatory elements (*35*). The CCCTC-binding factor (CTCF) binding sites generally organize local fixed nucleosome positioning. Further, the CTCF binding sites specifically contact other CTCF-binding sites at the boundaries of the larger TADs rather than enhancers (*35*) (Fig. 1A). This is often observed at a CTCF-binding site that was closely positioned to another nucleosome-free region, which resulted in fixed positioning of multiple nucleosomes relative to the DNA sequence (*35*) (Fig. 1A). These observations suggest that CTCF restricts the transcriptionally active regions to minimize energy consumption. This is in addition to the original concept that CTCF functions as an insulator-binding protein, which prevents enhancer–promoter interaction, leading to suppression of gene expression (*36–38*). CTCF also organizes extruding DNA into loops via cohesin, which belongs to the structural maintenance of chromosomes (SMC) family of ATPase complexes, generating TADs (*31–33*). Notably, ATP is required for TAD organization. In yeast ISW1a, an ATP-dependent nucleosome remodelling complex, slides nucleosomes into spaced arrays (Fig. 2) and interacts with cohesin (*39*). ISW1a is also thought to promote translocation of cohesin from the centromeric sites of loading to neighbouring regions (*39*). Therefore, repositioning nucleosomes by ATP-dependent chromatin remodelling complexes/ATPases may generate the nucleosome-free regions in conjunction with CTCF (Fig. 2) (described below). This system may be energetically efficient as ATP consumption is linked to interactions with the DNA-binding sites of CTCF. However, the DNA binding activity of TFs (but not their amount), which can act as a boundary limiting nucleosome sliding activity to one direction, is influenced by variations in DNA sequences in the flanking regions of their binding sites (*40**,*  *41*) (Fig. 2). This mechanism supports the concept of maintaining nucleosome-free regions by remodelling (sliding) complexes and explains the size differences of loops formed between enhancers and CTCFs (Figs 1A and 2).

Regarding biological significance, CTCF suppresses the somatic gene expression program and promotes a part of pluripotency-associated gene expression in mice (*36*). Importantly, SMARCA5, a subunit of the mouse ISWI complex, interacts with CTCF (*36**,*  *42*). Smarca5 knockdown leads to improper nucleosome positioning adjacent to CTCF-binding sites and reduces binding of CTCF and TFs at CTCF binding sites at the time when somatic cells are reprogrammed (*36*). Therefore, loss of SMARCA5 appears to reverse CTCF-insulator activity, maintaining somatic gene activation and impairing reprograming (*36*). The SWI/SNF ATP-dependent remodelling complex has been shown to bind to CTCF and localize on the loci overlapping with CTCF sites in human cells (*43*). Thus, SWI/SNF remodelling activity may influence CTCF activity and vice versa.

The reversible nature of chromatin structure commits signalling responses and epigenetic reprogramming. Nucleosome positioning becomes fuzzier in aged chromatin (*44*) as seen in lower nucleosome density in introns in late-passage compared to early-passage cultured human cells (*45*). It was shown that a low density of nucleosomes leads to increases in the speed of transcriptional elongation as measured by RNA polymerase II (Pol II) coverage during aging in five species including flies and humans. Moreover, reduction of elongation speeds by additional expression of nucleosomes extends lifespan in flies (*45*). Consistent with this, a reduction of histone H3Lys36 trimethylation (H3K36me3), a transcription elongation-linked histone modification, over open reading frames (ORFs) led to increased cryptic transcription and shorten replicative lifespan (RLS) in aged yeast cells and aged worms (*46*). H3K36me3 recruits the Rpd3S histone deacetylase complex and resets the nucleosomes with hypoacetylation and enriched H3K36me3 after Pol II passing in yeast (*47**,*  *48*). This may permit multiple rounds of proper transcription under reduced acetyl-CoA levels due to a decline in metabolism during aging. However, the downside is that lower nucleosome density and increased nucleosome displacement (histone exchange) during aging may expose cryptic promoters inducing cryptic transcription, which is the unintended initiation of transcription from non-promoter regions within the gene body, rather than the normal start site (*48–50*).

## Nucleotide metabolism may be behind altered chromatin substrates

DNA is sensitive to redox homeostasis, and its changes alter chromatin modification. DNA alkylating agents add alkyl groups to oxygen or nitrogen atoms in the DNA bases including modifying Ο6-guanine (Ο6G) to Ο6-methylguanine (Ο6meG) (*51*). Mismatch repair proteins, MSH2 and MSH6, form the MutSα complex to bind O6meG:C (*52*). O6meG:C can lead to mismatching generating O6meG:T. Unphosphorylated MSH6 binds O6meG, leading to MutSα binding O6meG-T mismatches, inducing DNA damage signalling by recruiting MutLα (MLH1-PMS2) (*52*). Stabilization of MutSα in response to alkylation damage occurs through interactions with the molybdopterin synthase associating complex (MPTAC), which regulates sulphur metabolism, and the Ada2a-containing histone acetyltransferase complex (ATAC) (*53**,*  *54*). These interactions have been lost in human lymphoblastoid cells from Fragile X-associated disorders (FXD) patients. FXD is caused by CGG repeat expansion in the 5′ untranslated region of the *FMR1* (*53–55*). Remarkably, these cells have altered purine metabolism (*56*), indicating that redox signalling relies on sulphur metabolism and purine metabolism to stabilize MutSα. Expansion of nucleotide repeats, such as CAG/CGG/ATTCT repeat, is a part of the pathogenesis of neurodegenerative disorders including Huntington’s disease (HD) (*57*). Base editing with G•C > A•T and A•T > G•C within CAG and GAA repeats alleles in the central nervous system of HD model mice reduced repeat expansion (*58*).

A recent study revealed that alteration of the cellular environment changes the length of tandem DNA repeats in somatic cells in a tissue-specific manner (*59*). An analysis of CAG trinucleotide repeats in blood cells using whole-genome sequencing (WGS) of biobank cohorts revealed that most human genomes contain unstable repeat alleles at 29 loci carrying repeat elements with somatic expansion (*59*). These allele-specific repeats were increased with aging although the instability of the expansions is common in the germline (*59*). Further search for genetic modifiers of repeat expansion found seven genetic modifiers of *TCF4* CAG repeat expansion including MSH3 and four genetic modifiers of non-CAG DNA repeats (more than three short tandem repeats), *MSH2*, *MSH3*, *MLH3* and *PMS2*, which encode mismatch repair proteins (*59*). Thus, an alkylating environment can be a high-risk factor for trinucleotide repeat disorders.

There are multiple links between redox status and chromatin modification. Notably, the human MSH2-MSH6 heterodimer disturbs a nucleosome (reducing affinity between DNA and histone octamer), and it was facilitated by histone H3 acetylation *in vivo* (*60*). Hence, altered redox homeostasis may alter chromatin formation within long range DNA regions including *cis*-regulatory elements associated with nucleotide repeat expansions. Interestingly, some nucleotide metabolic enzymes directly modify nucleosomes (*61–64*). For instance, guanosine 5′-monophosphate synthase (GMPS) deubiquitinates histone H2Bub1 through association with USP7, and phosphoribosylaminoimidazolesuccinocarboxamide (SAICAR) induces H3T11 phosphorylation through association with pyruvate kinase M2 (PKM2) (*61–64*). In addition, as ATP is highly utilized in DNA synthesis, the nucleotide metabolic enzymes may mediate a functional link between energy homeostasis and chromatin modifications in response to alteration of redox status.

## Metabolism Governs Chromatin Dynamics and Transcription for Redox Homeostasis

Chromatin modifiers establish chromatin dynamics by regulating DNA modifications and/or nucleosome modifications to achieve transcription, DNA replication, repair and recombination. Amino acids in the Ν-terminal tails of histones within nucleosomes are modified by attachment of functional groups of many types of acyls, methyl and phosphoryl moieties as well as ubiquitin, SUMO and others. This is termed post-translational modifications (PTMs) of histones (*65–67*). Most of these modifications are catalysed by chromatin modifiers, the majority of which form protein complexes that interact with DNA and/or nucleosomes and TFs and utilize cofactors in their enzymatic reactions (*3**,*  *11*). Distinct protein domains of histone modifying complex subunit(s) themselves recognize histone PTMs. Thus, histone modifying complexes recognizing histone modifications can lead to crosstalk/connections among PTMs (*65**,*  *66**,*  *68**,*  *69*). PTMs also influence how chromatin-modifying complexes, including ATP-dependent remodellers, bind to nucleosomes (*3**,*  *11**,*  *30**,*  *47**,*  *68**,*  *70–72*). Importantly, most of the cofactors for PTMs are metabolites (*3**,*  *73*). Therefore, the global levels of nucleosome PTMs may reflect metabolic status and energy availability. Moreover, ROS disrupt not only cofactor synthesis but also reactions of chromatin modifications in addition to damaging DNA (*5**,*  *12*). Here, we illustrate the effects of cellular metabolism on nucleosome structure and enzymatic activities by focusing on central metabolites. We also discuss roles of chromatin modification in metabolism.

### Acetyl-coenzyme A

Acetyl-coenzyme A (Acetyl-CoA) is a key metabolite from carbohydrate, amino acid, and fatty acid catabolism and is the substrate for histone acetylation (*74**,*  *75*). Histone acetyltransferases (HATs) use acetyl-CoA to transfer the acetyl group to the ε-amino group of lysine side chains of histones. Histone acetylation is thought to weaken the physical interactions between DNA and histones by neutralization of the positive charge on lysine, reducing the electrostatic interactions with the DNA backbone (*66**,*  *67*). A recent study using Debye-length Hamiltonian replica exchange molecular dynamics (D-HREMD) simulations illustrated that histone H3K27 acetylation leads to relaxed nucleosome positioning (*35*). This supports conventional concepts that nucleosome acetylation promotes transcription by increasing accessibility of the transcription activation machinery to the DNA. Histone acetyl-lysine is recognized by specific protein domains including bromodomains, YEATS (Yaf9, ENL, AF9, Taf14 and Sas5) domains and double plant homeodomain (PHD) fingers, each of which has distinct structure.

The global levels of histone acetylation in the nucleus are influenced by acetyl-CoA availability in cells. Therefore, glucose and mitochondrial respiration play critical roles in transcription regulation. In yeast, acetyl-CoA is utilized by the Spt-Ada-Gcn5-Acetyltransferase (SAGA) complex for the transcription activation of growth-promoting genes in the presence of glucose; however, in the absence of glucose, acetyl-CoA utilization is switched to mitochondrial ATP synthesis and formation of ketone bodies as alternative energy sources. Indeed, acetyl-CoA can be recaptured by deacetylation of histones on growth-promoting genes by the Rpd3 histone deacetylase complex (HDAC) (*50**,*  *76**,*  *77*).

The NAD^+^-dependent Sirtuins (SIRT) family contains seven class III histone deacetylases (*3*). Notably, SIRTs utilize NAD^+^ by activating the nicotinamide-ribose bond for nucleophilic attack in their deacylation reaction (*78*). This is different from the function of NAD^+^ as a redox cofactor. Increased cellular levels of NAD^+^ promotes Sir2-dependent gene repression in yeast (*79*). On the other hand, Sir2 suppresses genes associated with NAD^+^ biosynthesis (*80*). Thus, NAD^+^ and Sir2 are connected with a feedback loop. Reversible reactions between histone acetylation and deacetylation contribute to redox homeostasis through transcription regulation and protein modification involved in redox signalling.

In addition to acetylation, eight kinds of short-chain lysine (Lys) acylations on histones have been found. These include Lys propionylation (Kpr), Lys butyrylation (Kbu), Lys 2-hydroxyisobutyrylation (Khib), Lys succinylation (Ksucc), Lys malonylation (Kma), Lys glutarylation (Kglu), Lys crotonylation (Kcr) and Lys β-hydroxybutyrylation (Kbhb) (*81*). Acyl-group-specific transferases have not been identified. However, several acetyltransferases can also catalyse these alternative acylations (*81*). At least three HAT families: the Gcn5-related N-acetyltransferases (GNAT) family, the MOZ-, Ybf2/Sas3-, Sas2- and Tip60-related HATs (MYST family), and the p300/CBP HATs have been shown to catalyse Kpr (*67**,*  *81*). Histone deacylation is similar to histone deacetylation, some of the SIRTs and Zn2^+^-dependent HDACs have been found to catalyse removal of acyl groups with some specificities (*81**,*  *82*).

The recognition of Kpr, Kbu, Kcr and Ksucc in addition to Kac by bromodomains was comprehensively assessed by a crystal structure study using peptide binding (*83*). Interestingly, the preference of different acylations depends upon nutrition availability and determines which bromodomains can bind the acylation mark. A study using mice spermatogenic cells showed that both acetylation and butyrylation of histone H4K5 and H4K8 promote transcription; however, butyrylated H4K5 but not butyrylated H4K8 prevents binding of bromodomain 1 (BD1, essential binding domain) in the double bromodomain BET factor, Brdt (*84*). Since Brdt-BD1 directs the genome-wide replacement of histones (*85*), alternating acetylation vs butyrylation on H4K5 can change bromodomain-binding dynamics during spermatogenesis. Short-chain fatty acids have been suggested to be a source of acyl-CoA (*86*). Therefore, H4 acylation vs acetylation may alter upon a metabolic shift that changes the ratio of acyl-CoA/acetyl-CoA, or during a developmental event with an acyl-CoA requirement like during spermatogenesis (*87*).

### One-carbon

**Fig. 3:**
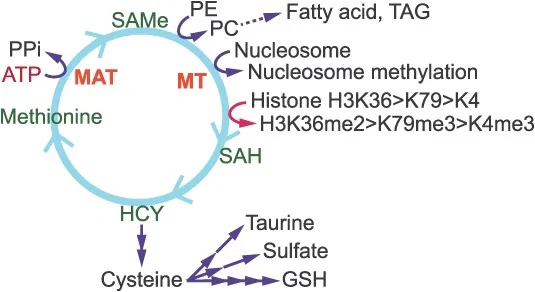
**The levels of methyl groups are regulated by coordination of nucleosomes, free histones and a phospholipid (PE).** The methionine cycle and cysteine metabolism pathway are regulated by chromatin modification and transcription. In normal growth conditions, methyl groups from SAMe are utilized predominantly for phospholipid methylation and then nucleosome methylation in the nucleus. Upon amino acid depletion, methyl groups are still utilized by phospholipids and nucleosomes; however, further excess methyl groups are stored on free histones catalysed by histone MTs. Histone methylation preferences are ranked as H3K36me2/3 > H3K27me3 > H3K4me3. Thus, free histones function as a methyl sink to prevent alkylation damage. SAMe, S-adenosyl-L-methionine; SAH, S-adenosyl-L-homocysteine; HCY, homocysteine; MAT, S-adenosylmethionine synthase, also methionine adenosyltransferase; MT, methyltransferase; PC, phosphatidylcholine; PE, phosphatidylethanolamine; TAG, triacylglycerol.

One-carbon (1C) metabolism mediated by folate redistributes carbon groups from inputs (usually serine and glycine) in nutrients (*88*). Outputs of 1C metabolism, nucleotides, glutathione (GSH), S-adenosyl-L-methionine (SAMe) and other metabolites, maintain cellular redox status (*88*) (Fig. 3). SAMe donates methyl groups to methyltransferases including lysine methyltransferases (KMTs), protein arginine methyltransferases (PRMTs) and histidine Nτ-methyltransferases. Methyl groups are transferred to DNA, lysine (K)/arginine (R)/histidine (H) residues in histones, splicing factors and other proteins (*54**,*  *73**,*  *89–93*). Both histone lysine and arginine methylation are associated with either activation or suppression of transcription dependent on the positions of the amino acid in the histone N-terminal tail in higher eukaryotes (*73**,*  *94*). Methylation of lysine residues occurs more frequently and is more abundant than methylation of arginine and histidine (*89*). Histone lysines can be mono-, di- or tri-methylated. Methyl-lysines are bound by distinct domains including chromodomain (CD), plant homeodomain (PHD) finger, Tudor, malignant brain tumour (MBT), proline-tryptophan-tryptophan-proline (PWWP), bromo adjacent homology (BAH), ankyrin repeats, WD40 repeats, ATRX-DNMT3A-DNMT3L (ADD) and zn-CW (*95*). There are three kinds of arginine methylation: monomethylarginine, asymmetric dimethylarginine and symmetric dimethylarginine (*96*). Tudor domains are the main readers of arginine methylation, and some of them bind both methyl-lysine and methyl-arginine (*96*).

Connections of 1C metabolism to the free radical scavenger system by free histone methylation and SWI/SNF are crucial for redox homeostasis. Methylation of a phospholipid, phosphatidylethanolamine (PE), is a major consumer of SAMe and promotes SAMe turnover for synthesis of cysteine and glutathione via transsulphuration in sulphur amino acid metabolism (Fig. 3). Sulphur metabolism functions as an oxygen-free radical scavenger (*13*) (Fig. 3). Interestingly, histone methylation, with a preference of H3K36 > H3K79 > H3K4, can also function as a methyl sink (*e.g.* consumes SAMe) to avoid detrimental damage from an excess of methyl groups upon loss of PE in yeast (*97*) (Fig. 3). SWI/SNF regulates transcription activation of sulphur metabolic genes by recruiting the sulphur-sensing TF Met4 at proper gene loci in yeast cultured in nutrient-rich media (*98*). Finally, as described above, destabilized MSH6 by dysfunction of sulphur metabolism has been suggested to underlie Fragile X disorders (*13**,*  *54*) (described in the ‘Nucleotide Metabolism’ section).

In mice, a methionine adenosyltransferase (MAT) isozyme, MATIIα, promotes H3K9me3 at the *COX-2* (*Cyclooxygenase-2*) loci by its interaction with the SETDB1 H3K9 methyltransferase (*99*) (Fig. 3). MATII synthesizes SAMe from methionine and ATP (*100*). Importantly, MafK is a transcription corepressor, which requires the catalytic activity of MATII for the transcription repression of genes including *HO1* (*heme oxygenase-1*). These target genes are oxidative stress-inducible genes (*101*). Thus, MATIIα suppresses oxidative stress responsive genes when nutrition is sufficient so that sulphur metabolism is not urgently required. It is not yet known whether MATII changes the methyltransferase partner(s) or changes target genes in conjunction with MafK in the absence of glucose.

Notably, cysteine(s) in nucleosomal histone H3 has been shown to be glutathionylated. Glutathionylation of H3 opens nucleosome structure (*102*). GSH is a major sulphur-containing antioxidant (*3**,*  *97**,*  *103*). It remains unclear whether antioxidant activity of glutathionylation on cysteine(s) affects modification of nearby lysines (*i.e.* methylation or acetylation).

### Oxygen

Oxygen availability directly modifies the catalytic activity of JmjC lysine demethylases (KDMs), which remove methyl groups from lysines within histone tails (*104**,*  *105*). JmjC are 2-oxoglutarate (2OG)/α-ketoglutarate-dependent oxygenases (2OGXs) and their reaction requires molecular oxygen and iron (II) (*104–106*). The oxygen dependency of H3K9me3 demethylation by KDM4E/JMJD2E was initially found *in vitro* (*107*). Subsequent studies revealed that demethylation of H3K9me2/3 and H3K36me2/3 by KDM4A/JMJD2A (*108*) observed under normoxia (21% O_2_) is suppressed upon hypoxia (1.0% O_2_) (*105*). KDM5A/JARID1 activity on H3K4me3/H3K36me3 and KDM6A/UTX activity on H3K27me3 follow the same patterns (*109**,*  *110*). 2OGX activity is dysregulated in cancer due to increased levels of metabolites from the tricarboxylic acid (TCA) cycle and presence of other metabolites, such as (R)-2-hydroxyglutarate (2HG) (*111**,*  *112*). Hypoxic environments at the inside of solid tumours may reduce 2OGX activity. Interestingly, hydroxylation of hypoxia inducible factor 1 subunit alpha (HIF1A) by prolyl hydroxylase domain enzymes destabilizes HIF1A and requires 2OG (*113*). 2-oxo acids have been suggested to selectively inhibit 2OG oxygenase activity (*112*). In addition to over expression of KDMs in cancer, such as KDM3B/JMJD1B, there are additional mechanisms by which KDMs contribute to oncogenesis (*114*). Thus, compounds, which are 2-oxo acid derivatives, may be applicable as enzyme inhibitors for treatment of cancers that have aberrant JmjC demethylase activities (*115*).

## Spatiotemporal Regulation of Chromatin Events Enables Cells to Minimize Cellular Energy Consumption

The involvement of liquid–liquid phase separation (LLPS) in transcription regulation and its possible effects on genome remodelling have been proposed (*5**,*  *116**,*  *117*). Classical phase separation of a homogeneous mixture is a process where the non-miscible two components separate by either nucleation and growth or spinodal decomposition until they form two different phases (*118*). There is viscoelastic phase separation that creates the dynamic asymmetry between two phases to form a network-like pattern of the slower phase composed with high-molecular-weight unstructured polymers (*e.g.* messenger RNA) (*119**,*  *120*).

Some *cis*-regulatory elements, enhancers, may be located far away from transcription start sites (TSSs). There is a class of enhancers called super enhancers (SEs), which are clustered with a high density of transcription and chromatin regulators. These can include RNA Pol II, general TFs, the bromodomain containing transcription coactivator p300 histone acetyltransferase, bromodomain containing 4 (BRD4), cohesin, the mediator complex, bidirectional transcripts (enhancer RNAs (eRNAs), a class of non-coding RNAs), acetylated histone H3K27 and multiple TFs (*117**,*  *121–124*) (Fig. 1B). These phenomena have been described as a phase separation model, and the effects of the phase separation on transcription have been studied. An increased concentration of transcriptional activators at SEs correlates with sigmoidal increases in the transcripts levels of SEs-corresponding genes (*117**,*  *121*). In addition, expression of eRNAs transcribed from enhancer regions (*123*) tightly correlates with transcription activation of corresponding active SEs (*117*). For instance, rapid and drastic *de novo* clustering of chromatin activators including accumulation of nuclear factor-kappa beta (NF-κΒ) (a TF) at enhancers (pre-existing enhancer motifs) creates inflammatory SEs upon tumour necrosis factor alpha (TNFα) stimulation and dominantly promotes inflammatory gene transcription. Meanwhile H3K27 acetylation is also increased at basal-endothelial-SEs in human endothelial cells (*125*). This might explain how asymmetric partitioning of different high-molecular-weight polymers can regulate the transcription levels.

Regarding the creation of phase separation, hyper H3K27ac at SEs suggests nucleosomes at SEs are relaxed. This is contrary to the notion that compaction of chromatin might execute better contributions to increases in the density of macromolecules at SEs. However, it may be explained by a model of enhancer-promoter contacts from 3C experiments suggesting that active nucleosome-free regions are extended above the chromatin surface (*35*) (described above) (Fig. 1B). Considering the 3C experiments, the total amount of nucleosome substrates for p300 must depend on the total number of *de novo* nucleosomes located on the chromatin surface at *cis*-regulatory elements. However, increased BRD4, whose bromodomains bind acetyl histone H3 and H4 in nucleosomes promiscuously (*126–128*), suggests that BRD4 mediates acetylation of nucleosomes over a broad region beyond those at *cis*-regulatory elements in SEs (Fig. 1B). A study of inflammatory SEs in human endothelial cells revealed that BRD4-bromodomains are crucial for the enhancer remodelling in the transition to inflammation (*125*), suggesting that nucleosome acetylation contributes to enhancer remodelling. Notably, the binding of BRD4 to p300-acetylated NF-κΒ promotes the transcription of NF-κΒ target genes in human cells (*129*). Another example of acetylation of TFs observed in SEs is MYC. MYC interacts with SEs through long-range interaction with eRNAs (*130**,*  *131*) and is acetylated by p300 and GCN5 in rat cells (*132*). Furthermore, eRNA directly interacts with the BRD4 bromodomain (*133*). Thus, acetylated non-histone molecules including TFs and eRNA may facilitate BRD4 recruitment, resulting in increases in the concentration of macromolecules (Fig. 1B). Importantly, many biomolecules participating in phase separation including p300 and NF-κΒ contain intrinsically disordered regions (IDRs). The multivalency of IDRs, which is the simultaneous binding of multiple ligands on one biological or chemical entity to multiple binding sites, often weak or dynamic, on another, can provide a platform for phase separation (*134–136*). In terms of energy homeostasis, phase separation may be an efficient method to achieve crucial events with minimum energy consumption within a minimum time period.

The contacts between distinct enhancers over long distances are established through nucleosome-free regions (*35*) (described above) (Fig. 1A). *In vitro* studies have found that BRD4 binding to acetylated lysine of human mononucleosomes requires flanking DNA (*128*). As BRD4 prefers binding nucleosomes with flanking DNA, it is possible that *de novo* creation of SEs may be initiated by BRD4 nucleosome binding and p300 nucleosome acetylation at the border of nucleosome-free regions before spreading acetylation. It may also require ATP-dependent chromatin remodelling to create nucleosome-free regions (Fig. 2).

## Future Perspective

Maintaining nucleosome-free regions, for instance blocking of nucleosome sliding via binding of TFs (Fig. 2), can be a key mechanism of chromatin dynamics for gene regulation. It also suggests a novel role of TFs in transcription regulation. Systematic analysis combining data on the binding affinity (dissociation constant) of TFs with natural sequences of TFs binding regions within chromatin and 3C analysis may predict the probability of enhancer–enhancer interactions and the probability of SEs. *In vitro* nucleosome remodelling assay can prove these sites and factors as barriers. There are also critical factors that change nucleosome structure, such as RNA Pol II (*137**,*  *138*) and natural G-rich sequences (*139*). Understanding the features of the interplay between chromatin dynamics and chromatin modifiers throughout the transcription activation process will reveal further mechanisms. Additionally, the energy requirements for a single transcription cycle may be measured. This will help quantify the relationships between chromatin dynamics, transcription and cellular metabolism.

## References

[1] Bartz, R.R. and Piantadosi, C.A. (2010) Clinical review: oxygen as a signaling molecule. Crit. Care 14, 234 10.1186/cc9185 PMC3219237 21062512

[2] Rigoulet, M., Bouchez, C.L., Paumard, P., Ransac, S., Cuvellier, S., Duvezin-Caubet, S., Mazat, J.P., and Devin, A. (2020) Cell energy metabolism: an update. Biochim. Biophys. Acta Bioenerg. 1861, 148276 10.1016/j.bbabio.2020.148276 32717222

[3] Suganuma, T. and Workman, J.L. (2018) Chromatin and metabolism. Annu. Rev. Biochem. 87, 27–49 10.1146/annurev-biochem-062917-012634 29925263

[4] Spinelli, J.B. and Haigis, M.C. (2018) The multifaceted contributions of mitochondria to cellular metabolism. Nat. Cell Biol. 20, 745–754 10.1038/s41556-018-0124-1 PMC6541229 29950572

[5] Suganuma, T. and Workman, J.L. (2023) Chromatin balances cell redox and energy homeostasis. Epigenet. Chromatin 16, 46 10.1186/s13072-023-00520-8 PMC10683155 38017471

[6] Valko, M., Leibfritz, D., Moncol, J., Cronin, M.T., Mazur, M., and Telser, J. (2007) Free radicals and antioxidants in normal physiological functions and human disease. Int. J. Biochem. Cell Biol. 39, 44–84 10.1016/j.biocel.2006.07.001 16978905

[7] Oh, S., Suganuma, T., Gogol, M.M., and Workman, J.L. (2018) Histone H3 threonine 11 phosphorylation by Sch9 and CK2 regulates chronological lifespan by controlling the nutritional stress response. eLife 7, 1–20 10.7554/eLife.36157 PMC6042962 29938647

[8] Pizzino, G., Irrera, N., Cucinotta, M., Pallio, G., Mannino, F., Arcoraci, V., Squadrito, F., Altavilla, D., and Bitto, A. (2017) Oxidative stress: harms and benefits for human health. Oxid. Med. Cell. Longev. 2017, 8416763 10.1155/2017/8416763 PMC5551541 28819546

[9] Mendez-Romero, O., Ricardez-Garcia, C., Castaneda-Tamez, P., Chiquete-Felix, N., and Uribe-Carvajal, S. (2022) Thriving in oxygen while preventing ROS overproduction: no two systems are created equal. Front. Physiol. 13, 874321 10.3389/fphys.2022.874321 PMC9013945 35444563

[10] Theriault, J.E., Shaffer, C., Dienel, G.A., Sander, C.Y., Hooker, J.M., Dickerson, B.C., Barrett, L.F., and Quigley, K.S. (2023) A functional account of stimulation-based aerobic glycolysis and its role in interpreting BOLD signal intensity increases in neuroimaging experiments. Neurosci. Biobehav. Rev. 153, 105373 10.1016/j.neubiorev.2023.105373 PMC10591873 37634556

[11] Church, M.C., Workman, J.L., and Suganuma, T. (2021) Macrophages, metabolites, and nucleosomes: chromatin at the intersection between aging and inflammation. Int. J. Mol. Sci. 22, 10274 10.3390/ijms221910274 PMC8508989 34638614

[12] Rubio, K., Hernandez-Cruz, E.Y., Rogel-Ayala, D.G., Sarvari, P., Isidoro, C., Barreto, G., and Pedraza-Chaverri, J. (2023) Nutriepigenomics in environmental-associated oxidative stress. Antioxidants 12, 771 10.3390/antiox12030771 PMC10045733 36979019

[13] Suganuma, T., Hassan, H., Swanson, S.K., Laxman, S., and Workman, J.L. (2026) Altered transposon element-derived genes distort oxygen-free radical scavenger systems in FXD. NAR Mol. Med. 3, ugag010 10.1093/narmme/ugag010 PMC12936792 41768973

[14] Buschbeck, M. and Hake, S.B. (2017) Variants of core histones and their roles in cell fate decisions, development and cancer. Nat. Rev. Mol. Cell Biol. 18, 299–314 10.1038/nrm.2016.166 28144029

[15] Marzluff, W.F., Wagner, E.J., and Duronio, R.J. (2008) Metabolism and regulation of canonical histone mRNAs: life without a poly(a) tail. Nat. Rev. Genet. 9, 843–854 10.1038/nrg2438 PMC2715827 18927579

[16] Groth, A., Rocha, W., Verreault, A., and Almouzni, G. (2007) Chromatin challenges during DNA replication and repair. Cell 128, 721–733 10.1016/j.cell.2007.01.030 17320509

[17] Vincent, J.A., Kwong, T.J., and Tsukiyama, T. (2008) ATP-dependent chromatin remodeling shapes the DNA replication landscape. Nat. Struct. Mol. Biol. 15, 477–484 10.1038/nsmb.1419 PMC2678716 18408730

[18] Clapier, C.R., Iwasa, J., Cairns, B.R., and Peterson, C.L. (2017) Mechanisms of action and regulation of ATP-dependent chromatin-remodelling complexes. Nat. Rev. Mol. Cell Biol. 18, 407–422 10.1038/nrm.2017.26 PMC8127953 28512350

[19] Bartholomew, B. (2014) Regulating the chromatin landscape: structural and mechanistic perspectives. Annu. Rev. Biochem. 83, 671–696 10.1146/annurev-biochem-051810-093157 PMC4332854 24606138

[20] Narlikar, G.J., Sundaramoorthy, R., and Owen-Hughes, T. (2013) Mechanisms and functions of ATP-dependent chromatin-remodeling enzymes. Cell 154, 490–503 10.1016/j.cell.2013.07.011 PMC3781322 23911317

[21] Becker, P.B. and Workman, J.L. (2013) Nucleosome remodeling and epigenetics. Cold Spring Harb. Perspect. Biol. 5, a017905 10.1101/cshperspect.a017905 PMC3753709 24003213

[22] Owen-Hughes, T., Utley, R.T., Cote, J., Peterson, C.L., and Workman, J.L. (1996) Persistent site-specific remodeling of a nucleosome array by transient action of the SWI/SNF complex. Science 273, 513–516 10.1126/science.273.5274.513 8662543

[23] Cote, J., Quinn, J., Workman, J.L., and Peterson, C.L. (1994) Stimulation of GAL4 derivative binding to nucleosomal DNA by the yeast SWI/SNF complex. Science 265, 53–60 10.1126/science.8016655 8016655

[24] Yamada, K., Frouws, T.D., Angst, B., Fitzgerald, D.J., DeLuca, C., Schimmele, K., Sargent, D.F., and Richmond, T.J. (2011) Structure and mechanism of the chromatin remodelling factor ISW1a. Nature 472, 448–453 10.1038/nature09947 21525927

[25] Deindl, S., Hwang, W.L., Hota, S.K., Blosser, T.R., Prasad, P., Bartholomew, B., and Zhuang, X. (2013) ISWI remodelers slide nucleosomes with coordinated multi-base-pair entry steps and single-base-pair exit steps. Cell 152, 442–452 10.1016/j.cell.2012.12.040 PMC3647478 23374341

[26] Vary, J.C., Jr., Gangaraju, V.K., Qin, J., Landel, C.C., Kooperberg, C., Bartholomew, B., and Tsukiyama, T. (2003) Yeast Isw1p forms two separable complexes *in vivo*. Mol. Cell. Biol. 23, 80–91 10.1128/MCB.23.1.80-91.2003 PMC140669 12482963

[27] Hada, A., Hota, S.K., Luo, J., Lin, Y.C., Kale, S., Shaytan, A.K., Bhardwaj, S.K., Persinger, J., Ranish, J., Panchenko, A.R., and Bartholomew, B. (2019) Histone octamer structure is altered early in ISW2 ATP-dependent nucleosome remodeling. Cell Rep. 28, e286 10.1016/j.celrep.2019.05.106 31269447

[28] Saha, A., Wittmeyer, J., and Cairns, B.R. (2005) Chromatin remodeling through directional DNA translocation from an internal nucleosomal site. Nat. Struct. Mol. Biol. 12, 747–755 10.1038/nsmb973 16086025

[29] Clapier, C.R., Kasten, M.M., Parnell, T.J., Viswanathan, R., Szerlong, H., Sirinakis, G., Zhang, Y., and Cairns, B.R. (2016) Regulation of DNA translocation efficiency within the chromatin remodeler RSC/Sth1 potentiates nucleosome sliding and ejection. Mol. Cell 62, 453–461 10.1016/j.molcel.2016.03.032 PMC5291166 27153540

[30] Suganuma, T., Gutierrez, J.L., Li, B., Florens, L., Swanson, S.K., Washburn, M.P., Abmayr, S.M., and Workman, J.L. (2008) ATAC is a double histone acetyltransferase complex that stimulates nucleosome sliding. Nat. Struct. Mol. Biol. 15, 364–372 10.1038/nsmb.1397 18327268

[31] Davidson, I.F., Barth, R., Zaczek, M., van der Torre, J., Tang, W., Nagasaka, K., Janissen, R., Kerssemakers, J., Wutz, G., Dekker, C., and Peters, J.M. (2023) CTCF is a DNA-tension-dependent barrier to cohesin-mediated loop extrusion. Nature 616, 822–827 10.1038/s41586-023-05961-5 PMC10132984 37076620

[32] Nora, E.P., Goloborodko, A., Valton, A.L., Gibcus, J.H., Uebersohn, A., Abdennur, N., Dekker, J., Mirny, L.A., and Bruneau, B.G. (2017) Targeted degradation of CTCF decouples local insulation of chromosome domains from genomic compartmentalization. Cell 169, e922 10.1016/j.cell.2017.05.004 PMC5538188 28525758

[33] Kong, M., Cutts, E.E., Pan, D., Beuron, F., Kaliyappan, T., Xue, C., Morris, E.P., Musacchio, A., Vannini, A., and Greene, E.C. (2020) Human Condensin I and II drive extensive ATP-dependent compaction of nucleosome-bound DNA. Mol. Cell 79, e119 10.1016/j.molcel.2020.04.026 PMC7335352 32445620

[34] Dixon, J.R., Selvaraj, S., Yue, F., Kim, A., Li, Y., Shen, Y., Hu, M., Liu, J.S., and Ren, B. (2012) Topological domains in mammalian genomes identified by analysis of chromatin interactions. Nature 485, 376–380 10.1038/nature11082 PMC3356448 22495300

[35] Li, H., Dalgleish, J.L.T., Lister, G., Maristany, M.J., Huertas, J., Dopico-Fernandez, A.M., Hamley, J.C., Denny, N., Bloye, G., Zhang, W., Hentges, L., Doll, R., Wei, Y., Maresca, M., Dimitrova, E., Pytowski, L., Tunnacliffe, E.A.J., Kassouf, M., Higgs, D., de Wit, E., Klose, R.J., Schermelleh, L., Collepardo-Guevara, R., Milne, T.A., and Davies, J.O.J. (2025) Mapping chromatin structure at base-pair resolution unveils a unified model of cis-regulatory element interactions. Cell 188, e7119 10.1016/j.cell.2025.10.013 PMC7618578 41197626

[36] Song, Y., Liang, Z., Zhang, J., Hu, G., Wang, J., Li, Y., Guo, R., Dong, X., Babarinde, I.A., Ping, W., Sheng, Y.L., Li, H., Chen, Z., Gao, M., Chen, Y., Shan, G., Zhang, M.Q., Hutchins, A.P., Fu, X.D., and Yao, H. (2022) CTCF functions as an insulator for somatic genes and a chromatin remodeler for pluripotency genes during reprogramming. Cell Rep. 39, 110626 10.1016/j.celrep.2022.110626 35385732

[37] Chen, D., Keremane, S., Wang, S., and Lei, E.P. (2025) CTCF regulates global chromatin accessibility and transcription during rod photoreceptor development. Proc. Natl. Acad. Sci. U.S.A. 122, e2416384122 10.1073/pnas.2416384122 PMC11892594 39993185

[38] Bell, A.C., West, A.G., and Felsenfeld, G. (1999) The protein CTCF is required for the enhancer blocking activity of vertebrate insulators. Cell 98, 387–396 10.1016/s0092-8674(00)81967-4 10458613

[39] Litwin, I., Nowicka, M., Markowska, K., Maciaszczyk-Dziubinska, E., Tomaszewska, P., Wysocki, R., and Kramarz, K. (2023) ISW1a modulates cohesin distribution in centromeric and pericentromeric regions. Nucleic Acids Res. 51, 9101–9121 10.1093/nar/gkad612 PMC10516642 37486771

[40] Raiqueo, F., Amigo, R., and Gutierrez, J.L. (2025) Variations in flanking or less conserved positions of Reb1 and Abf1 consensus binding sites lead to major changes in their ability to modulate nucleosome sliding activity. Biol. Res. 58, 53 10.1186/s40659-025-00627-0 PMC12305957 40731031

[41] Amigo, R., Raiqueo, F., Tarifeno, E., Farkas, C., and Gutierrez, J.L. (2023) Poly(dA:dT) tracts differentially modulate nucleosome remodeling activity of RSC and ISW1a complexes, exerting tract orientation-dependent and -independent effects. Int. J. Mol. Sci. 24, 15245 10.3390/ijms242015245 PMC10607297 37894925

[42] Wiechens, N., Singh, V., Gkikopoulos, T., Schofield, P., Rocha, S., and Owen-Hughes, T. (2016) The chromatin remodelling enzymes SNF2H and SNF2L position nucleosomes adjacent to CTCF and other transcription factors. PLoS Genet. 12, e1005940 10.1371/journal.pgen.1005940 PMC4809547 27019336

[43] Valletta, M., Russo, R., Baglivo, I., Russo, V., Ragucci, S., Sandomenico, A., Iaccarino, E., Ruvo, M., De Feis, I., Angelini, C., Iachettini, S., Biroccio, A., Pedone, P.V., and Chambery, A. (2020) Exploring the interaction between the SWI/SNF chromatin remodeling complex and the zinc finger factor CTCF. Int. J. Mol. Sci. 21, 8950 10.3390/ijms21238950 PMC7728349 33255744

[44] Hu, Z., Chen, K., Xia, Z., Chavez, M., Pal, S., Seol, J.H., Chen, C.C., Li, W., and Tyler, J.K. (2014) Nucleosome loss leads to global transcriptional up-regulation and genomic instability during yeast aging. Genes Dev. 28, 396–408 10.1101/gad.233221.113 PMC3937517 24532716

[45] Debes, C., Papadakis, A., Gronke, S., Karalay, O., Tain, L.S., Mizi, A., Nakamura, S., Hahn, O., Weigelt, C., Josipovic, N., Zirkel, A., Brusius, I., Sofiadis, K., Lamprousi, M., Lu, Y.X., Huang, W., Esmaillie, R., Kubacki, T., Späth, M.R., Schermer, B., Benzing, T., Müller, R.U., Antebi, A., Partridge, L., and Papantonis, A. (2023) Ageing-associated changes in transcriptional elongation influence longevity. Nature 616, 814–821 10.1038/s41586-023-05922-y PMC10132977 37046086

[46] Sen, P., Dang, W., Donahue, G., Dai, J., Dorsey, J., Cao, X., Liu, W., Cao, K., Perry, R., Lee, J.Y., Wasko, B.M., Carr, D.T., He, C., Robison, B., Wagner, J., Gregory, B.D., Kaeberlein, M., Kennedy, B.K., Boeke, J.D., and Berger, S.L. (2015) H3K36 methylation promotes longevity by enhancing transcriptional fidelity. Genes Dev. 29, 1362–1376 10.1101/gad.263707.115 PMC4511212 26159996

[47] Venkatesh, S., Smolle, M., Li, H., Gogol, M.M., Saint, M., Kumar, S., Natarajan, K., and Workman, J.L. (2012) Set2 methylation of histone H3 lysine 36 suppresses histone exchange on transcribed genes. Nature 489, 452–455 10.1038/nature11326 22914091

[48] Workman, J.L. (2006) Nucleosome displacement in transcription. Genes Dev. 20, 2009–2017 10.1101/gad.1435706 16882978

[49] Venkatesh, S., Li, H., Gogol, M.M., and Workman, J.L. (2016) Selective suppression of antisense transcription by Set2-mediated H3K36 methylation. Nat. Commun. 7, 13610 10.1038/ncomms13610 PMC5133703 27892455

[50] Carrozza, M.J., Li, B., Florens, L., Suganuma, T., Swanson, S.K., Lee, K.K., Shia, W.J., Anderson, S., Yates, J., Washburn, M.P., and Workman, J.L. (2005) Histone H3 methylation by Set2 directs deacetylation of coding regions by Rpd3S to suppress spurious intragenic transcription. Cell 123, 581–592 10.1016/j.cell.2005.10.023 16286007

[51] Soll, J.M., Sobol, R.W., and Mosammaparast, N. (2017) Regulation of DNA alkylation damage repair: lessons and therapeutic opportunities. Trends Biochem. Sci. 42, 206–218 10.1016/j.tibs.2016.10.001 PMC5336464 27816326

[52] Kaliyaperumal, S., Patrick, S.M., and Williams, K.J. (2011) Phosphorylated hMSH6: DNA mismatch versus DNA damage recognition. Mutat. Res. 706, 36–45 10.1016/j.mrfmmm.2010.10.008 PMC3010429 21035467

[53] Suganuma, T. and Workman, J.L. (2022) MPTAC links alkylation damage signaling to sterol biosynthesis. Redox Biol. 51, 102270 10.1016/j.redox.2022.102270 PMC8866156 35189552

[54] Suganuma, T., Swanson, S.K., Gogol, M., Garrett, T.J., Conkright-Fincham, J., Florens, L., Washburn, M.P., and Workman, J.L. (2018) MPTAC determines APP fragmentation via sensing sulfur amino acid catabolism. Cell Rep. 24, 1585–1596 10.1016/j.celrep.2018.07.013 30089268

[55] Suganuma, T., Gutierrez, J.L., Li, B., Florens, L., Swanson, S.K., Washburn, M.P., Abmayr, S.M., and Workman, J.L. (2008) ATAC is a double histone acetyltransferase complex that stimulates nucleosome sliding. Nat. Struct. Mol. Biol. 15, 364–372 10.1038/nsmb.1397 18327268

[56] Suganuma, T., Hassan, H., Gogol, M., and Workman, J.L. (2024) C G composition in transposon-derived genes is increased in FXD with perturbed immune system. NAR Mol. Medicine 1, ugae015 10.1093/narmme/ugae015 PMC11500580 39465205

[57] Orr, H.T. and Zoghbi, H.Y. (2007) Trinucleotide repeat disorders. Annu. Rev. Neurosci. 30, 575–621 10.1146/annurev.neuro.29.051605.113042 17417937

[58] Matuszek, Z., Arbab, M., Kesavan, M., Hsu, A., Roy, J.C.L., Zhao, J., Yu, T., Weisburd, B., Newby, G.A., Doherty, N.J., Wu, M., Shibata, S., Cristian, A., Tao, Y.A., Fearnley, L.G., Bahlo, M., Rehm, H.L., Xie, J., Gao, G., Mouro Pinto, R., and Liu, D.R. (2025) Base editing of trinucleotide repeats that cause Huntington's disease and Friedreich's ataxia reduces somatic repeat expansions in patient cells and in mice. Nat. Genet. 57, 1437–1451 10.1038/s41588-025-02172-8 PMC12165863 40419681

[59] Hujoel, M.L.A., Handsaker, R.E., Tang, D., Kamitaki, N., Mukamel, R.E., Rubinacci, S., Palamara, P.F., McCarroll, S.A., and Loh, P.R. (2026) Insights into DNA repeat expansions among 900,000 biobank participants. Nature 650, 920–929 10.1038/s41586-025-09886-z PMC12935551 41501457

[60] Javaid, S., Manohar, M., Punja, N., Mooney, A., Ottesen, J.J., Poirier, M.G., and Fishel, R. (2009) Nucleosome remodeling by hMSH2-hMSH6. Mol. Cell 36, 1086–1094 10.1016/j.molcel.2009.12.010 PMC3010363 20064472

[61] van der Knaap, J.A., Kumar, B.R., Moshkin, Y.M., Langenberg, K., Krijgsveld, J., Heck, A.J., Karch, F., and Verrijzer, C.P. (2005) GMP synthetase stimulates histone H2B deubiquitylation by the epigenetic silencer USP7. Mol. Cell 17, 695–707 10.1016/j.molcel.2005.02.013 15749019

[62] Reddy, B.A., van der Knaap, J.A., Bot, A.G., Mohd-Sarip, A., Dekkers, D.H., Timmermans, M.A., Martens, J.W., Demmers, J.A., and Verrijzer, C.P. (2014) Nucleotide biosynthetic enzyme GMP synthase is a TRIM21-controlled relay of p53 stabilization. Mol. Cell 53, 458–470 10.1016/j.molcel.2013.12.017 24462112

[63] Keller, K.E., Tan, I.S., and Lee, Y.S. (2012) SAICAR stimulates pyruvate kinase isoform M2 and promotes cancer cell survival in glucose-limited conditions. Science 338, 1069–1072 10.1126/science.1224409 PMC3527123 23086999

[64] Suganuma, T. and Workman, J.L. (2021) Nucleotide metabolism behind epigenetics. Front. Endocrinol. 12, 731648 10.3389/fendo.2021.731648 PMC8435732 34526971

[65] Suganuma, T. and Workman, J.L. (2008) Crosstalk among histone modifications. Cell 135, 604–607 10.1016/j.cell.2008.10.036 19013272

[66] Suganuma, T. and Workman, J.L. (2011) Signals and combinatorial functions of histone modifications. Annu. Rev. Biochem. 80, 473–499 10.1146/annurev-biochem-061809-175347 21529160

[67] Dutta, A., Abmayr, S.M., and Workman, J.L. (2016) Diverse activities of histone acylations connect metabolism to chromatin function. Mol. Cell 63, 547–552 10.1016/j.molcel.2016.06.038 PMC5298895 27540855

[68] Li, S., Swanson, S.K., Gogol, M., Florens, L., Washburn, M.P., Workman, J.L., and Suganuma, T. (2015) Serine and SAM responsive complex SESAME regulates histone modification crosstalk by sensing cellular metabolism. Mol. Cell 60, 408–421 10.1016/j.molcel.2015.09.024 26527276

[69] Bian, C., Xu, C., Ruan, J., Lee, K.K., Burke, T.L., Tempel, W., Barsyte, D., Li, J., Wu, M., Zhou, B.O., Fleharty, B.E., Paulson, A., Allali-Hassani, A., Zhou, J.Q., Mer, G., Grant, P.A., Workman, J.L., Zang, J., and Min, J. (2011) Sgf29 binds histone H3K4me2/3 and is required for SAGA complex recruitment and histone H3 acetylation. EMBO J. 30, 2829–2842 10.1038/emboj.2011.193 PMC3160252 21685874

[70] Utley, R.T., Ikeda, K., Grant, P.A., Cote, J., Steger, D.J., Eberharter, A., John, S., and Workman, J.L. (1998) Transcriptional activators direct histone acetyltransferase complexes to nucleosomes. Nature 394, 498–502 10.1038/28886 9697775

[71] Grant, P.A., Schieltz, D., Pray-Grant, M.G., Steger, D.J., Reese, J.C., Yates, J.R., 3rd, and Workman, J.L. (1998) A subset of TAF(II)s are integral components of the SAGA complex required for nucleosome acetylation and transcriptional stimulation. Cell 94, 45–53 10.1016/s0092-8674(00)81220-9 9674426

[72] Kusch, T., Florens, L., Macdonald, W.H., Swanson, S.K., Glaser, R.L., Yates, J.R., 3rd, Abmayr, S.M., Washburn, M.P., and Workman, J.L. (2004) Acetylation by Tip60 is required for selective histone variant exchange at DNA lesions. Science 306, 2084–2087 10.1126/science.1103455 15528408

[73] Etchegaray, J.P. and Mostoslavsky, R. (2016) Interplay between metabolism and epigenetics: a nuclear adaptation to environmental changes. Mol. Cell 62, 695–711 10.1016/j.molcel.2016.05.029 PMC4893201 27259202

[74] Shi, L. and Tu, B.P. (2015) Acetyl-CoA and the regulation of metabolism: mechanisms and consequences. Curr. Opin. Cell Biol. 33, 125–131 10.1016/j.ceb.2015.02.003 PMC4380630 25703630

[75] Lee, K.K. and Workman, J.L. (2007) Histone acetyltransferase complexes: one size doesn't fit all. Nat. Rev. Mol. Cell Biol. 8, 284–295 10.1038/nrm2145 17380162

[76] Hsieh, W.C., Sutter, B.M., Ruess, H., Barnes, S.D., Malladi, V.S., and Tu, B.P. (2022) Glucose starvation induces a switch in the histone acetylome for activation of gluconeogenic and fat metabolism genes. Mol. Cell 82, e65 10.1016/j.molcel.2021.12.015 PMC8794035 34995509

[77] Grant, P.A., Duggan, L., Cote, J., Roberts, S.M., Brownell, J.E., Candau, R., Ohba, R., Owen-Hughes, T., Allis, C.D., Winston, F., Berger, S.L., and Workman, J.L. (1997) Yeast Gcn5 functions in two multisubunit complexes to acetylate nucleosomal histones: characterization of an Ada complex and the SAGA (Spt/Ada) complex. Genes Dev. 11, 1640–1650 10.1101/gad.11.13.1640 9224714

[78] Sauve, A.A. (2010) Sirtuin chemical mechanisms. Biochim. Biophys. Acta 1804, 1591–1603 10.1016/j.bbapap.2010.01.021 PMC2886189 20132909

[79] Belenky, P., Racette, F.G., Bogan, K.L., McClure, J.M., Smith, J.S., and Brenner, C. (2007) Nicotinamide riboside promotes Sir2 silencing and extends lifespan via Nrk and Urh1/Pnp1/Meu1 pathways to NAD+. Cell 129, 473–484 10.1016/j.cell.2007.03.024 17482543

[80] Humphrey, K.M., Zhu, L., Hickman, M.A., Hasan, S., Maria, H., Liu, T., and Rusche, L.N. (2020) Evolution of distinct responses to low NAD(+) stress by rewiring the Sir2 deacetylase network in yeasts. Genetics 214, 855–868 10.1534/genetics.120.303087 PMC7153948 32071196

[81] Sabari, B.R., Zhang, D., Allis, C.D., and Zhao, Y. (2017) Metabolic regulation of gene expression through histone acylations. Nat. Rev. Mol. Cell Biol. 18, 90–101 10.1038/nrm.2016.140 PMC5320945 27924077

[82] Rousseaux, S. and Khochbin, S. (2015) Histone acylation beyond acetylation: terra incognita in chromatin biology. Cell J. 17, 1–6 10.22074/cellj.2015.506 PMC4393657 25870829

[83] Flynn, E.M., Huang, O.W., Poy, F., Oppikofer, M., Bellon, S.F., Tang, Y., and Cochran, A.G. (2015) A subset of human bromodomains recognizes butyryllysine and crotonyllysine histone peptide modifications. Structure 23, 1801–1814 10.1016/j.str.2015.08.004 26365797

[84] Goudarzi, A., Zhang, D., Huang, H., Barral, S., Kwon, O.K., Qi, S., Tang, Z., Buchou, T., Vitte, A.L., He, T., Cheng, Z., Montellier, E., Gaucher, J., Curtet, S., Debernardi, A., Charbonnier, G., Puthier, D., Petosa, C., Panne, D., Rousseaux, S., Roeder, R.G., Zhao, Y., and Khochbin, S. (2016) Dynamic competing histone H4 K5K8 acetylation and butyrylation are hallmarks of highly active gene promoters. Mol. Cell 62, 169–180 10.1016/j.molcel.2016.03.014 PMC4850424 27105113

[85] Gaucher, J., Boussouar, F., Montellier, E., Curtet, S., Buchou, T., Bertrand, S., Hery, P., Jounier, S., Depaux, A., Vitte, A.L., Guardiola, P., Pernet, K., Debernardi, A., Lopez, F., Holota, H., Imbert, J., Wolgemuth, D.J., Gérard, M., Rousseaux, S., and Khochbin, S. (2012) Bromodomain-dependent stage-specific male genome programming by Brdt. EMBO J. 31, 3809–3820 10.1038/emboj.2012.233 PMC3463845 22922464

[86] Xie, Z., Zhang, D., Chung, D., Tang, Z., Huang, H., Dai, L., Qi, S., Li, J., Colak, G., Chen, Y., Xia, C., Peng, C., Ruan, H., Kirkey, M., Wang, D., Jensen, L.M., Kwon, O.K., Lee, S., Pletcher, S.D., Tan, M., Lombard, D.B., White, K.P., Zhao, H., Li, J., Roeder, R.G., Yang, X., and Zhao, Y. (2016) Metabolic regulation of gene expression by histone lysine beta-hydroxybutyrylation. Mol. Cell 62, 194–206 10.1016/j.molcel.2016.03.036 PMC5540445 27105115

[87] Schonfeld, P. and Wojtczak, L. (2016) Short- and medium-chain fatty acids in energy metabolism: the cellular perspective. J. Lipid Res. 57, 943–954 10.1194/jlr.R067629 PMC4878196 27080715

[88] Shuvalov, O., Petukhov, A., Daks, A., Fedorova, O., Vasileva, E., and Barlev, N.A. (2017) One-carbon metabolism and nucleotide biosynthesis as attractive targets for anticancer therapy. Oncotarget 8, 23955–23977 10.18632/oncotarget.15053 PMC5410357 28177894

[89] Hayashi, T., Daitoku, H., Uetake, T., Kako, K., and Fukamizu, A. (2023) Histidine Nτ-methylation identified as a new posttranslational modification in histone H2A at His-82 and H3 at His-39. J. Biol. Chem. 299, 105131 10.1016/j.jbc.2023.105131 PMC10485160 37543365

[90] Johnson, P., Harris, C.I., and Perry, S.V. (1967) 3-methylhistidine in actin and other muscle proteins. Biochem. J. 105, 361–370 10.1042/bj1050361 PMC1198308 6056634

[91] Lin, W.J., Gary, J.D., Yang, M.C., Clarke, S., and Herschman, H.R. (1996) The mammalian immediate-early TIS21 protein and the leukemia-associated BTG1 protein interact with a protein-arginine N-methyltransferase. J. Biol. Chem. 271, 15034–15044 10.1074/jbc.271.25.15034 8663146

[92] Kwiatkowski, S., Seliga, A.K., Vertommen, D., Terreri, M., Ishikawa, T., Grabowska, I., Tiebe, M., Teleman, A.A., Jagielski, A.K., Veiga-da-Cunha, M., and Drozak, J. (2018) SETD3 protein is the actin-specific histidine N-methyltransferase. eLife 7, e37921 10.7554/eLife.37921 PMC6289574 30526847

[93] Webb, K.J., Zurita-Lopez, C.I., Al-Hadid, Q., Laganowsky, A., Young, B.D., Lipson, R.S., Souda, P., Faull, K.F., Whitelegge, J.P., and Clarke, S.G. (2010) A novel 3-methylhistidine modification of yeast ribosomal protein Rpl3 is dependent upon the YIL110W methyltransferase. J. Biol. Chem. 285, 37598–37606 10.1074/jbc.M110.170787 PMC2988365 20864530

[94] Blanc, R.S. and Richard, S. (2017) Arginine methylation: the coming of age. Mol. Cell 65, 8–24 10.1016/j.molcel.2016.11.003 28061334

[95] Wilkinson, A.W. and Gozani, O. (2014) Histone-binding domains: strategies for discovery and characterization. Biochim. Biophys. Acta 1839, 669–675 10.1016/j.bbagrm.2014.01.007 PMC4099300 24525102

[96] Gayatri, S. and Bedford, M.T. (2014) Readers of histone methylarginine marks. Biochim. Biophys. Acta 1839, 702–710 10.1016/j.bbagrm.2014.02.015 PMC4099268 24583552

[97] Ye, C., Sutter, B.M., Wang, Y., Kuang, Z., and Tu, B.P. (2017) A metabolic function for phospholipid and histone methylation. Mol. Cell 66, e188 10.1016/j.molcel.2017.02.026 PMC5482412 28366644

[98] Church, M.C., Price, A., Li, H., and Workman, J.L. (2023) The Swi-Snf chromatin remodeling complex mediates gene repression through metabolic control. Nucleic Acids Res. 51, 10278–10291 10.1093/nar/gkad711 PMC10602859 37650639

[99] Kera, Y., Katoh, Y., Ohta, M., Matsumoto, M., Takano-Yamamoto, T., and Igarashi, K. (2013) Methionine adenosyltransferase II-dependent histone H3K9 methylation at the COX-2 gene locus. J. Biol. Chem. 288, 13592–13601 10.1074/jbc.M112.429738 PMC3650394 23539621

[100] Sanderson, S.M., Gao, X., Dai, Z., and Locasale, J.W. (2019) Methionine metabolism in health and cancer: a nexus of diet and precision medicine. Nat. Rev. Cancer 19, 625–637 10.1038/s41568-019-0187-8 31515518

[101] Katoh, Y., Ikura, T., Hoshikawa, Y., Tashiro, S., Ito, T., Ohta, M., Kera, Y., Noda, T., and Igarashi, K. (2011) Methionine adenosyltransferase II serves as a transcriptional corepressor of Maf oncoprotein. Mol. Cell 41, 554–566 10.1016/j.molcel.2011.02.018 21362551

[102] Garcia-Gimenez, J.L., Olaso, G., Hake, S.B., Bonisch, C., Wiedemann, S.M., Markovic, J., Dasi, F., Gimeno, A., Perez-Quilis, C., Palacios, O., Capdevila, M., Vina, J., and Pallardo, F.V. (2013) Histone H3 glutathionylation in proliferating mammalian cells destabilizes nucleosomal structure. Antioxid. Redox Signal. 19, 1305–1320 10.1089/ars.2012.5021 PMC3791047 23541030

[103] Fujii, J., Osaki, T., Soma, Y., and Matsuda, Y. (2023) Critical roles of the cysteine-glutathione axis in the production of gamma-glutamyl peptides in the nervous system. Int. J. Mol. Sci. 24, 8044 10.3390/ijms24098044 PMC10179188 37175751

[104] Tsukada, Y., Fang, J., Erdjument-Bromage, H., Warren, M.E., Borchers, C.H., Tempst, P., and Zhang, Y. (2006) Histone demethylation by a family of JmjC domain-containing proteins. Nature 439, 811–816 10.1038/nature04433 16362057

[105] Hancock, R.L., Masson, N., Dunne, K., Flashman, E., and Kawamura, A. (2017) The activity of JmjC histone lysine demethylase KDM4A is highly sensitive to oxygen concentrations. ACS Chem. Biol. 12, 1011–1019 10.1021/acschembio.6b00958 PMC5404277 28051298

[106] Frost, J., Frost, M., Batie, M., Jiang, H., and Rocha, S. (2021) Roles of HIF and 2-oxoglutarate-dependent dioxygenases in controlling gene expression in hypoxia. Cancers 13, 350 10.3390/cancers13020350 PMC7832865 33477877

[107] Sanchez-Fernandez, E.M., Tarhonskaya, H., Al-Qahtani, K., Hopkinson, R.J., McCullagh, J.S., Schofield, C.J., and Flashman, E. (2013) Investigations on the oxygen dependence of a 2-oxoglutarate histone demethylase. Biochem. J. 449, 491–496 10.1042/BJ20121155 PMC4673901 23092293

[108] Couture, J.F., Collazo, E., Ortiz-Tello, P.A., Brunzelle, J.S., and Trievel, R.C. (2007) Specificity and mechanism of JMJD2A, a trimethyllysine-specific histone demethylase. Nat. Struct. Mol. Biol. 14, 689–695 10.1038/nsmb1273 17589523

[109] Batie, M., Frost, J., Frost, M., Wilson, J.W., Schofield, P., and Rocha, S. (2019) Hypoxia induces rapid changes to histone methylation and reprograms chromatin. Science 363, 1222–1226 10.1126/science.aau5870 30872526

[110] Chakraborty, A.A., Laukka, T., Myllykoski, M., Ringel, A.E., Booker, M.A., Tolstorukov, M.Y., Meng, Y.J., Meier, S.R., Jennings, R.B., Creech, A.L., Herbert, Z.T., McBrayer, S.K., Olenchock, B.A., Jaffe, J.D., Haigis, M.C., Beroukhim, R., Signoretti, S., Koivunen, P., and Kaelin, W.G., Jr. (2019) Histone demethylase KDM6A directly senses oxygen to control chromatin and cell fate. Science 363, 1217–1222 10.1126/science.aaw1026 PMC7336390 30872525

[111] Losman, J.A., Koivunen, P., and Kaelin, W.G., Jr. (2020) 2-oxoglutarate-dependent dioxygenases in cancer. Nat. Rev. Cancer 20, 710–726 10.1038/s41568-020-00303-3 33087883

[112] Nakashima, Y., Brewitz, L., Tumber, A., Salah, E., and Schofield, C.J. (2021) 2-oxoglutarate derivatives can selectively enhance or inhibit the activity of human oxygenases. Nat. Commun. 12, 6478 10.1038/s41467-021-26673-2 PMC8580996 34759269

[113] Burr, S.P., Costa, A.S., Grice, G.L., Timms, R.T., Lobb, I.T., Freisinger, P., Dodd, R.B., Dougan, G., Lehner, P.J., Frezza, C., and Nathan, J.A. (2016) Mitochondrial protein lipoylation and the 2-oxoglutarate dehydrogenase complex controls HIF1α stability in aerobic conditions. Cell Metab. 24, 740–752 10.1016/j.cmet.2016.09.015 PMC5106373 27923773

[114] Manni, W., Jianxin, X., Weiqi, H., Siyuan, C., and Huashan, S. (2022) JMJD family proteins in cancer and inflammation. Signal Transduct. Target. Ther. 7, 304 10.1038/s41392-022-01145-1 PMC9434538 36050314

[115] Breski, M., Dey, D., Obringer, S., Sudhamalla, B., and Islam, K. (2016) Engineering biological C-H functionalization leads to allele-specific regulation of histone demethylases. J. Am. Chem. Soc. 138, 13505–13508 10.1021/jacs.6b08653 27709909

[116] Hansen, J.C., Maeshima, K., and Hendzel, M.J. (2021) The solid and liquid states of chromatin. Epigenet. Chromatin 14, 50 10.1186/s13072-021-00424-5 PMC8557566 34717733

[117] Hah, N., Benner, C., Chong, L.W., Yu, R.T., Downes, M., and Evans, R.M. (2015) Inflammation-sensitive super enhancers form domains of coordinately regulated enhancer RNAs. Proc. Natl. Acad. Sci. U.S.A. 112, E297–E302 10.1073/pnas.1424028112 PMC4311831 25564661

[118] Fernandez-Rico, C., Sai, T., Sicher, A., Style, R.W., and Dufresne, E.R. (2022) Putting the squeeze on phase separation. JACS Au 2, 66–73 10.1021/jacsau.1c00443 PMC8790737 35098222

[119] Erkamp, N.A., Sneideris, T., Ausserwoger, H., Qian, D., Qamar, S., Nixon-Abell, J., St George-Hyslop, P., Schmit, J.D., Weitz, D.A., and Knowles, T.P.J. (2023) Spatially non-uniform condensates emerge from dynamically arrested phase separation. Nat. Commun. 14, 684 10.1038/s41467-023-36059-1 PMC9908939 36755024

[120] Tanaka, H. (2022) Viscoelastic phase separation in biological cells. Commun. phys. 5, 1–12

[121] Hnisz, D., Shrinivas, K., Young, R.A., Chakraborty, A.K., and Sharp, P.A. (2017) A phase separation model for transcriptional control. Cell 169, 13–23 10.1016/j.cell.2017.02.007 PMC5432200 28340338

[122] Hah, N., Danko, C.G., Core, L., Waterfall, J.J., Siepel, A., Lis, J.T., and Kraus, W.L. (2011) A rapid, extensive, and transient transcriptional response to estrogen signaling in breast cancer cells. Cell 145, 622–634 10.1016/j.cell.2011.03.042 PMC3099127 21549415

[123] Wang, Y., Zhang, C., Wang, Y., Liu, X., and Zhang, Z. (2022) Enhancer RNA (eRNA) in human diseases. Int. J. Mol. Sci. 23, 11582 10.3390/ijms231911582 PMC9569849 36232885

[124] Allen, B.L. and Taatjes, D.J. (2015) The Mediator complex: a central integrator of transcription. Nat. Rev. Mol. Cell Biol. 16, 155–166 10.1038/nrm3951 PMC4963239 25693131

[125] Brown, J.D., Lin, C.Y., Duan, Q., Griffin, G., Federation, A., Paranal, R.M., Bair, S., Newton, G., Lichtman, A., Kung, A., Yang, T., Wang, H., Luscinskas, F.W., Croce, K.J., Bradner, J.E., and Plutzky, J. (2014) NF-κB directs dynamic super enhancer formation in inflammation and atherogenesis. Mol. Cell 56, 219–231 10.1016/j.molcel.2014.08.024 PMC4224636 25263595

[126] Dey, A., Chitsaz, F., Abbasi, A., Misteli, T., and Ozato, K. (2003) The double bromodomain protein Brd4 binds to acetylated chromatin during interphase and mitosis. Proc. Natl. Acad. Sci. U.S.A. 100, 8758–8763 10.1073/pnas.1433065100 PMC166386 12840145

[127] Jung, M., Philpott, M., Muller, S., Schulze, J., Badock, V., Eberspacher, U., Moosmayer, D., Bader, B., Schmees, N., Fernandez-Montalvan, A., and Haendler, B. (2014) Affinity map of bromodomain protein 4 (BRD4) interactions with the histone H4 tail and the small molecule inhibitor JQ1. J. Biol. Chem. 289, 9304–9319 10.1074/jbc.M113.523019 PMC3979416 24497639

[128] Lambrechts, L.S., Reid, X.J., Kambanis, L., Luong, C., Kobakhidze, E., Daners, A., Zhong, Y., Patel, K., Franck, C.K., Sani, H.M., Taylor, C., Low, J.K.K., Payne, R.J., and Mackay, J.P. (2025) The BRD4-nucleosome interaction is enhanced modestly and non-selectively by histone acetylation. Nucleic Acids Res. 53, gkaf1406 10.1093/nar/gkaf1406 PMC12746106 41459750

[129] Huang, B., Yang, X.D., Zhou, M.M., Ozato, K., and Chen, L.F. (2009) Brd4 coactivates transcriptional activation of NF-κB via specific binding to acetylated RelA. Mol. Cell. Biol. 29, 1375–1387 10.1128/MCB.01365-08 PMC2643823 19103749

[130] Pomerantz, M.M., Ahmadiyeh, N., Jia, L., Herman, P., Verzi, M.P., Doddapaneni, H., Beckwith, C.A., Chan, J.A., Hills, A., Davis, M., Yao, K., Kehoe, S.M., Lenz, H.J., Haiman, C.A., Yan, C., Henderson, B.E., Frenkel, B., Barretina, J., Bass, A., Tabernero, J., Baselga, J., Regan, M.M., Manak, J.R., Shivdasani, R., Coetzee, G.A., and Freedman, M.L. (2009) The 8q24 cancer risk variant rs6983267 shows long-range interaction with MYC in colorectal cancer. Nat. Genet. 41, 882–884 10.1038/ng.403 PMC2763485 19561607

[131] Grisanzio, C. and Freedman, M.L. (2010) Chromosome 8q24-associated cancers and MYC. Genes Cancer 1, 555–559 10.1177/1947601910381380 PMC3092220 21779458

[132] Hurd, M., Pino, J., Jang, K., Allevato, M.M., Vorontchikhina, M., Ichikawa, W., Zhao, Y., Gates, R., Villalpando, E., Hamilton, M.J., Faiola, F., Pan, S., Qi, Y., Hung, Y.W., Girke, T., Ann, D., Seewaldt, V., and Martinez, E. (2023) MYC acetylated lysine residues drive oncogenic cell transformation and regulate select genetic programs for cell adhesion-independent growth and survival. Genes Dev. 37, 865–882 10.1101/gad.350736.123 PMC10691474 37852796

[133] Rahnamoun, H., Lee, J., Sun, Z., Lu, H., Ramsey, K.M., Komives, E.A., and Lauberth, S.M. (2018) RNAs interact with BRD4 to promote enhanced chromatin engagement and transcription activation. Nat. Struct. Mol. Biol. 25, 687–697 10.1038/s41594-018-0102-0 PMC6859054 30076409

[134] Holehouse, A.S. and Kragelund, B.B. (2024) The molecular basis for cellular function of intrinsically disordered protein regions. Nat. Rev. Mol. Cell Biol. 25, 187–211 10.1038/s41580-023-00673-0 PMC11459374 37957331

[135] Ibrahim, Z., Wang, T., Destaing, O., Salvi, N., Hoghoughi, N., Chabert, C., Rusu, A., Gao, J., Feletto, L., Reynoird, N., Schalch, T., Zhao, Y., Blackledge, M., Khochbin, S., and Panne, D. (2022) Structural insights into p300 regulation and acetylation-dependent genome organisation. Nat. Commun. 13, 7759 10.1038/s41467-022-35375-2 PMC9755262 36522330

[136] Ghosh, G., Wang, V.Y., Huang, D.B., and Fusco, A. (2012) NF-κB regulation: lessons from structures. Immunol. Rev. 246, 36–58 10.1111/j.1600-065X.2012.01097.x PMC4543363 22435546

[137] Kujirai, T., Ehara, H., Fujino, Y., Shirouzu, M., Sekine, S.I., and Kurumizaka, H. (2018) Structural basis of the nucleosome transition during RNA polymerase II passage. Science 362, 595–598 10.1126/science.aau9904 30287617

[138] Ehara, H., Kujirai, T., Fujino, Y., Shirouzu, M., Kurumizaka, H., and Sekine, S.I. (2019) Structural insight into nucleosome transcription by RNA polymerase II with elongation factors. Science 363, 744–747 10.1126/science.aav8912 30733384

[139] Varshney, D., Spiegel, J., Zyner, K., Tannahill, D., and Balasubramanian, S. (2020) The regulation and functions of DNA and RNA G-quadruplexes. Nat. Rev. Mol. Cell Biol. 21, 459–474 10.1038/s41580-020-0236-x PMC7115845 32313204

